# Cognitive Control Deficits in Alcohol Dependence Are a Trait- and State-Dependent Biomarker: An ERP Study

**DOI:** 10.3389/fpsyt.2020.606891

**Published:** 2020-12-08

**Authors:** Xiaohong Liu, Hongliang Zhou, Chenguang Jiang, Yanling Xue, Zhenhe Zhou, Jun Wang

**Affiliations:** ^1^Department of Substance Dependence, The Affiliated Wuxi Mental Health Center of Nanjing Medical University, Wuxi, China; ^2^Nanjing Brain Hospital Affiliated to Nanjing Medical University, Wuxi, China; ^3^Department of Psychiatry, The Affiliated Wuxi Mental Health Center of Nanjing Medical University, Wuxi, China

**Keywords:** alcohol dependence, cognitive control, event-related potential, trait dependent biomarker, state dependent biomarker

## Abstract

Alcohol dependence (AD) presents cognitive control deficits. Event-related potential (ERP) P300 reflects cognitive control-related processing. The aim of this study was to investigate whether cognitive control deficits are a trait biomarker or a state biomarker in AD. Participants included 30 AD patients and 30 healthy controls (HCs). All participants were measured with P300 evoked by a three-stimulus auditory oddball paradigm at a normal state (time 1, i.e., just after the last alcohol intake) and abstinence (time 2, i.e., just after a 4-week abstinence). The results showed that for P3a and P3b amplitude, the interaction effect for group × time point was significant, the simple effect for group at time 1 level and time 2 level was significant, and the simple effect for time point at AD group level was significant; however, the simple effect for time point at HC group level was not significant. Above results indicated that compared to HCs, AD patients present reductions of P3a/3b amplitude, and after 4-week alcohol abstinence, although P3a/3b amplitudes were improved, they were still lower than those of HCs. For P3a and P3b latencies, no significant differences were observed. These findings conclude that AD patients present cognitive control deficits that are reflected by P3a/3b and that cognitive control deficits in AD are trait- and state-dependent. The implication of these findings is helpful to understand the psychological and neural processes for AD, and these findings suggest that improving the cognitive control function may impact the treatment effect for AD.

## Introduction

Alcohol dependence (AD) is a relapsing disorder and presents a loss of volitional control over consumption, impaired executive functions, a pathological preoccupation with alcohol seeking, and a compulsive drive for harmful drinking, disregarding many serious life consequences, such as deteriorating health, professional responsibilities, and family loss. AD involves not only alcohol-related liver and cerebral cortex diseases but also violence and traffic accidents. Understanding the psychological and neural processes of AD is an important public health issue.

Cognitive control is a sort of cognitive ability that is involved in the adjustment of perceptual selection and action; namely, cognitive control can be regarded as a flexible, goal-directed behavior that is essential for efficient information processing and behavioral response under conditions of uncertainty and underlies a broad range of executive functions (1, 2). AD is associated with cognitive control dysfunctions, and cognitive control is mediated through the interaction between inherent large-scale brain networks involved in externally oriented executive functioning and internally focused thought processing (3). Many previous studies have indicated that AD patients present cognitive control dysfunction; in particular, altered impulse control has been implicated in AD. For example, a study used functional magnetic resonance imaging (fMRI) during a stop signal task to investigate cognitive control function in AD patients, and the results showed that AD patients displayed longer go trial and stop signal reaction times, a higher stop success rate and post-error slowing; AD patients displayed less activity in cortical and subcortical structures, including the putamen, insula, and amygdala, during risk-taking decisions in the stop signal task. These results provided evidence for altered neural processing during impulse control in AD (4). A recent study used fMRI to investigate the relationship between AD severity and delay discounting neural activation and concluded that AD severity tracks with dysregulations in cognitive control and reward evaluation areas during impulsive and delayed decisions (5). Another study also used fMRI to investigate the relationship between AD severity and functional connectivity of fronto-striatal networks during a stop signal task, and the results indicated that patients with more severe AD displayed less frontal connectivity with the striatum, a component of cognitive control networks important for response inhibition (6).

Event-related potential (ERP) is a tool for a functional measure of brain activity that occurs time-locked to external stimuli and reflects successive stages of information processing. The ERP is a technique that can provide an analysis of neural activity with the high temporal resolution and high informative power on neural alterations in several disorders including schizophrenia, affective disorders, and substance abuse disorders (7–9). Previous studies have indicated that ERP P300 (P300) reflects cognitive control-related processing; therefore, P300 is thought to serve as a marker of cognitive control processes (10, 11). P300 includes P3a and P3b. P3b is elicited by target stimuli of the traditional oddball task, whereas P3a is elicited by novel or nontarget stimuli of the traditional oddball task. Substantial evidence exists regarding P300 deficits in AD patients. However, whether P3a/3b deficits are present primarily during AD (i.e., state-dependent) or are an integral part of the disorder (i.e., trait-dependent) is still controversial. Many studies have confirmed that AD patients displayed reduced P3a/3b amplitudes while performing an oddball task (12), suggesting that AD patients exhibited a disability to allocate neural resources for encoding specific stimuli, which could be due to impaired cortical functions. These results support P300 deficit as a trait biomarker in AD. However, a study showed that P300 deficit was not present in treatment-naive alcohol-dependent patients without comorbidities (13), which support the concept that P300 might be a state-dependent biomarker in AD. Clarifying whether a cognitive control deficit, which is reflected by P3a/3b deficits, is a trait biomarker or a state biomarker in AD will be helpful to understand the psychological and neural processes for AD.

Considering that a state characteristic is transient and a trait characteristic is enduring (14), longitudinal research is essential to determine whether P3a/3b deficits are state- or trait-dependent in AD. No longitudinal study to date has investigated whether P3a/3b deficits either are present primarily during AD (i.e., state-dependent) or form an integral part of the disorder (i.e., trait-dependent), or a combination of the two (i.e., state- as well as trait-dependent).

Alcohol-dependent patients' cognitive problem is also an emotion-anxiety problem. Previous studies showed that children of alcohol-dependent patients displayed a higher frequency of psychopathological states, which are known to have a strong effect on depression or anxiety (15), and present altered activations of the amygdala, which is involved in the elicitation and decoding of emotional feeling (16). The State-Trait Anxiety Inventory (STAI) has a sensitivity in detecting anxiety disorders and anxiety-like behaviors (17) and has been employed to capture enduring characteristics and patterns of symptoms (18). However, the assessment of the anxiety level by using STAI depends on the participant's ability to subjectively comment or report on his or her own mental state (18). Therefore, using the measurement of P3a/3b to determine whether cognitive control deficits are trait- and state-dependent can be an advantage of objectivity of the assessment of anxiety level in AD.

The Severity of Alcohol Dependence Questionnaire (SADQ) is usually used for the assessment of the severity of AD (19). A previous study showed that the Chinese version of the Severity of Alcohol Dependence Questionnaire (SADQ-C) consists of four principal components, including withdrawal relief drinking, affective withdrawal signs, physical withdrawal signs, and reinstatement of withdrawal symptoms following abstinence. The internal consistency of SADQ-C was Cronbach's α of 0.92, which confirmed that the SADQ-C is a reliable tool for AD severity assessment and it can be used to administer the treatment outcome in male patients with AD (20).

In the present study, patients with AD were selected as subjects, and cognitive control functions were measured with P3a and P3b, which were elicited by a three-stimulus oddball task; assessments of cognitive control functions were performed at baseline and after a 4-week follow-up. The hypothesis of this study is that cognitive control deficits in AD are both a trait- and state-dependent biomarker, which is reflected by P3a/3b. The aim of this study was to investigate whether a cognitive control deficit, which is reflected by P3a/3b deficits, is a trait biomarker or a state biomarker in AD.

## Methods

### Time and Setting

The present study was conducted in the Department of Substance Dependence, The Affiliated Wuxi Mental Health Centre of Nanjing Medical University, China, from March 1, 2018, to April 30, 2020.

### Diagnostic Criteria and Participants

The present study included an AD group and a healthy control (HC) group. The criteria for inclusion in the AD group were as follows: (1) met the Diagnostic and Statistical Manual of Mental Disorders, Fifth Edition (DSM-5), criteria for AD; (2) were in an age range from 18 to 60 years old; (3) did not receive any medication for 2 weeks prior to the study; (4) were not smokers; and (5) had no neurological illness or comorbid psychiatric illness, as determined by medical records, or other substance dependence. The inclusion criteria for the HC group were as follows: (1) did not meet the criteria for any DSM-5 axis I disorder or personality disorders, as assessed by the Structured Clinical Interview for DSM-5 (SCID-5, Chinese version); (2) were in an age range from 18 to 60 years old; and (3) had no history of any kind of mental disorder or any kind of physical illness.

In this study, 30 AD patients were recruited as the AD group. The AD patients were inpatients at the Department of Substance Dependence. Thirty healthy individuals were recruited as the HC group. HCs were recruited from a group of citizens who lived in Wuxi City, Jiangsu Province, China, through local advertising. Both AD patients and HCs were Chinese.

### Event-Related Potential Measurement

**Figure 1:**
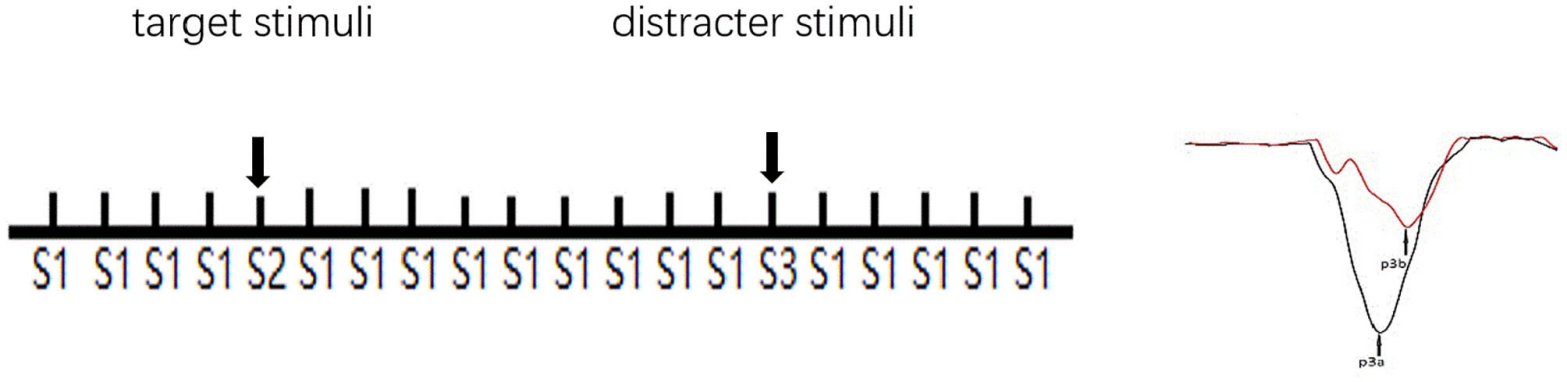
Schematic illustration of the three-stimulus auditory oddball paradigm. S1: standard stimuli. S2: infrequent target stimuli randomly appeared in a background of frequently occurring S1. S3: infrequent distracter stimuli randomly appeared in a background of occurring S1. The subjects were instructed to respond (click the left mouse button) when S2, but not any other stimuli, appeared. S3 elicits a P3a, and S2 elicits a P3b.

ERP measurement was taken from a recent study (21). The BioSemi Active Two system (BioSemi Inc., Amsterdam, Netherlands) was employed continuously for the electroencephalogram (EEG) record. The digitization rate was 512 Hertz (Hz); the bandpass was DC-104 Hz, and the common mode sense served as the reference (PO2 site) using a 64-channel electrode cap. Electro-oculogram electrodes were placed below and at the outer canthi of the left eye. A three-stimulus auditory oddball paradigm was employed to elicit P3a and P3b. A total of 400 binaural, 80-decibel (dB) tones with 50-ms-duration stimuli were presented to the participants through foam insert earphones. Overall, 12% of the stimuli were target tones (1,500 Hz), 12% infrequent “novel” sounds (a bird call or a water drop), and 76% standard tones (1,000 Hz), with an interstimulus interval varying between 1.8 and 2.2 s. Stimuli presentation was randomized. The electrical impedance was monitored. The duration of the whole P300 paradigm is 8 min. Participants were in a sound attenuated chamber. All subjects were told to press the computer mouse button in response to the target tones. Clicking occurrence between 100 and 900 ms after the tone served as a correct response. Before the formal trial, there was a practice block to make sure participants understood the task (see Figure 1).

### Event-Related Potential Data Analysis

Brain Vision Analyzer 2.0 (Brain Products GmbH, Munich, Germany) was used for ERP data analysis. P3a was analyzed at the Cz site because it is largest in the frontal regions, and P3b was analyzed at the Pz site because it is largest over the parietal regions (21–23). An average of the mastoids was the reference and was bandpass filtered between 0.01 and 20 Hz using a zero-phase shift Butterworth filter. Data were segmented by a stimulus marker from −100 to 1,000 ms, responses to novel sounds were employed for P3a, and correct responses to target tones were employed for P3b. Segments were baseline-corrected using a −100 to 0 ms pre-stimulus time and eye-blink corrected using established measures. Artifact rejection for individual channels was performed, and a given segment was rejected if the voltage gradient exceeded 50 μV/ms, the amplitude was ±100 μV, or the signal was flat (<0.5 μV for more than 100 ms). Segments were averaged across stimulus markers, and the P3a amplitude peak was chosen from 250 to 450 ms, while the P3b amplitude peak was chosen from 280 to 650 ms.

### Experiment Procedures

On the day of the ERP recording, two psychiatric resident physicians collected patient demographic data, clinical characteristics, and confirmed/excluded a diagnosis of AD. The Annett handedness scale was used for the assessments of handedness (24). AD levels were measured with the SADQ, and a breath alcohol reading was used to measure blood alcohol concentration in the AD group.

All AD patients were measured with P300 at a normal state (time 1, i.e., just after the last alcohol intake) and abstinence (time 2, i.e., just after a 4-week abstinence). When measuring P300 at time 2, all AD patients had to end any treatment with medication for 2 weeks. To avoid the practice effect, the HCs were measured with P300 twice in a 2-week interval (corresponding to time 1 and time 2). The anxiety and depression of all participants were assessed with the Hamilton Anxiety Scale (HAMA) and the Hamilton Depression Scale (17-item edition, HAMD).

All experimental procedures were approved by the Ethics Committee on Human Studies, the Affiliated Wuxi Mental Health Centre of Nanjing Medical University, Wuxi, Jiangsu Province, China, and they were conducted in accordance with the Declaration of Helsinki. All participants provided their written informed consent to participate, and all were compensated with 600.00 Chinese Yuan plus travel costs.

### Data Analysis

Statistical Program for the Social Sciences software version 19.0 (SPSS, IBM Corporation, Armonk, NY, USA) was employed for the data analysis. Mean age and education were compared between the AD group and the HC group using two-tailed *t*-tests, and handedness was compared using the Pearson chi-square test. HAMD and HAMA scores were compared between the AD group and the HC group using paired-samples *t-*tests. The mean amplitudes and the mean latencies of P3a and P3b were compared between the AD group and the HC group using repeated ANOVA. The degrees of freedom of the *F* ratio were corrected according to the Greenhouse–Geisser method. Least square difference tests were performed as *post hoc* analyses if indicated. Alpha values of 0.05 were considered significant.

## Results

### Demographic Characteristics of Participants

Demographic characteristics of participants are shown in Table 1. There were no significant differences between the demographic characteristics of the participants in the AD group and those of the people in the HC group. The HAMA and HAMD scores were higher in the AD group than in the HC group; however, no significant differences were observed.

**Table 1:** Demographic and clinical characteristics of participants.

	**AD**	**HC**	**Test statistic**
Sex ratio (M/F)	30/0	30/0	–
Mean age (SD), years	43.2 (7.4)	43.9 (7.6)	*t* = 0.110, *p* = 0.912
Age range	27–57	28–59	–
Education (SD)	8.3 (2.1)	9.4 (2.1)	*t* = 1.763, *p* = 0.850
Years of addiction (SD)	18.9 (10.0)	–	–
Handedness (R/M/L)	10/9/11	11/10/9	χ2 = 0.230, *p* = 0.725
SADQ (SD)	26.4 (2.5)	–	–
Blood alcohol concentration (mg/100 ml, SD)	75.6 (3.2)	–	–
HAMA (SD)	5.6 (1.3)	3.5 (1.9)	*t* = 0.241, *p* = 0.621
HAMD (SD)	6.4 (2.3)	5.1 (2.5)	*t* = 0.107, *p* = 0.735

*AD, alcohol-dependent individual group; HC, normal control group; M, male; F, female; SD, standard deviation; R, right; M, mixed; L, left; SADQ, Severity of Alcohol Dependence Questionnaire; HAMD, Hamilton Depression Scale; HAMA, Hamilton Anxiety Scale*.

### Event-Related Potential Data Analysis

ERP data from the AD group and the HC group are shown in Table 2. In this study, using P3a and P3b as dependent variables, a 2 × 2 repeated-measures ANOVA on mean amplitudes and the mean latencies with group (AD group vs. HC group) as a between-subjects factor and time point (time 1 vs. time 2) as a within-subjects factor, was performed.

**Table 2:** ERP data [mean (SD)] in the AD group (*n* = 30) and HC group (*n* = 30).

**Variable**	**AD (time 1)**		**AD (time 2)**		**HC (time 1)**		**HC (time 2)**	
	**A (μV)**	**L (ms)**	**A (μV)**	**L (ms)**	**A (μV)**	**L (ms)**	**A (μV)**	**L (ms)**
P3a	6.6 (1.6)	310.7 (18.3)	8.6 (1.0)	308.5 (24.9)	9.9 (0.6)	302.2 (25.7)	9.7 (0.9)	307.0 (29.6)
P3b	4.4 (1.5)	354.7 (29.6)	5.9 (1.5)	351.5 (24.1)	7.2 (1.3)	350.5 (24.2)	7.0 (1.2)	349.3 (24.5)

*AD, alcohol dependence; HC, health control; SD, standard deviation; A, amplitude; L, latency; ERP, event-related potential*.

#### P3a Component

**Figure 2:**
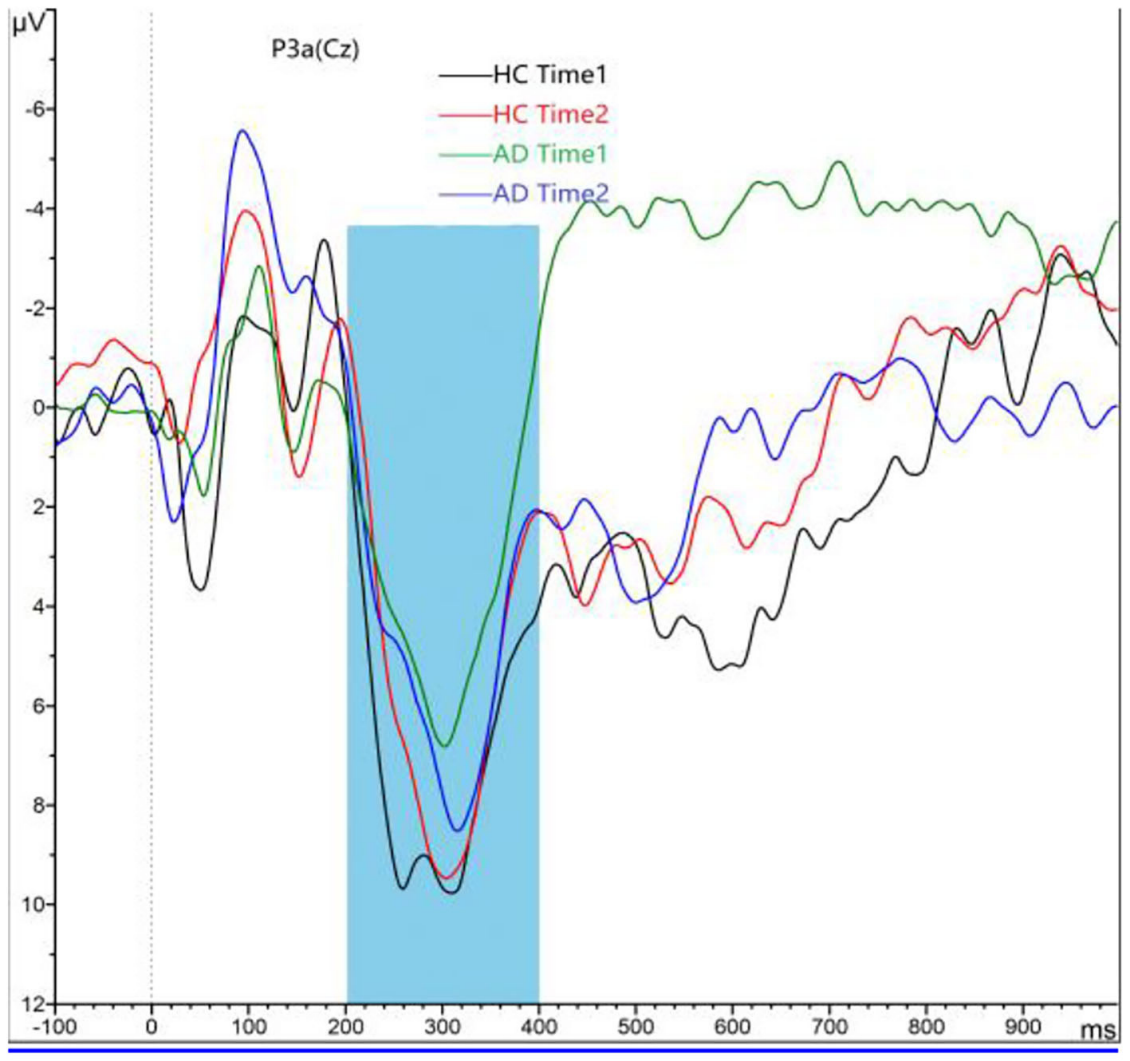
Grand averaged P3a was elicited by a three-stimulus auditory oddball paradigm for the alcohol dependence (AD) group (purple lines and green lines) and the healthy control (HC) group (black lines and red lines) at time 1 and time 2. The P3a was presented within a 250- to 450-ms latency window at the Cz electrode site. The gray area is timeframe.

**Figure 3:**
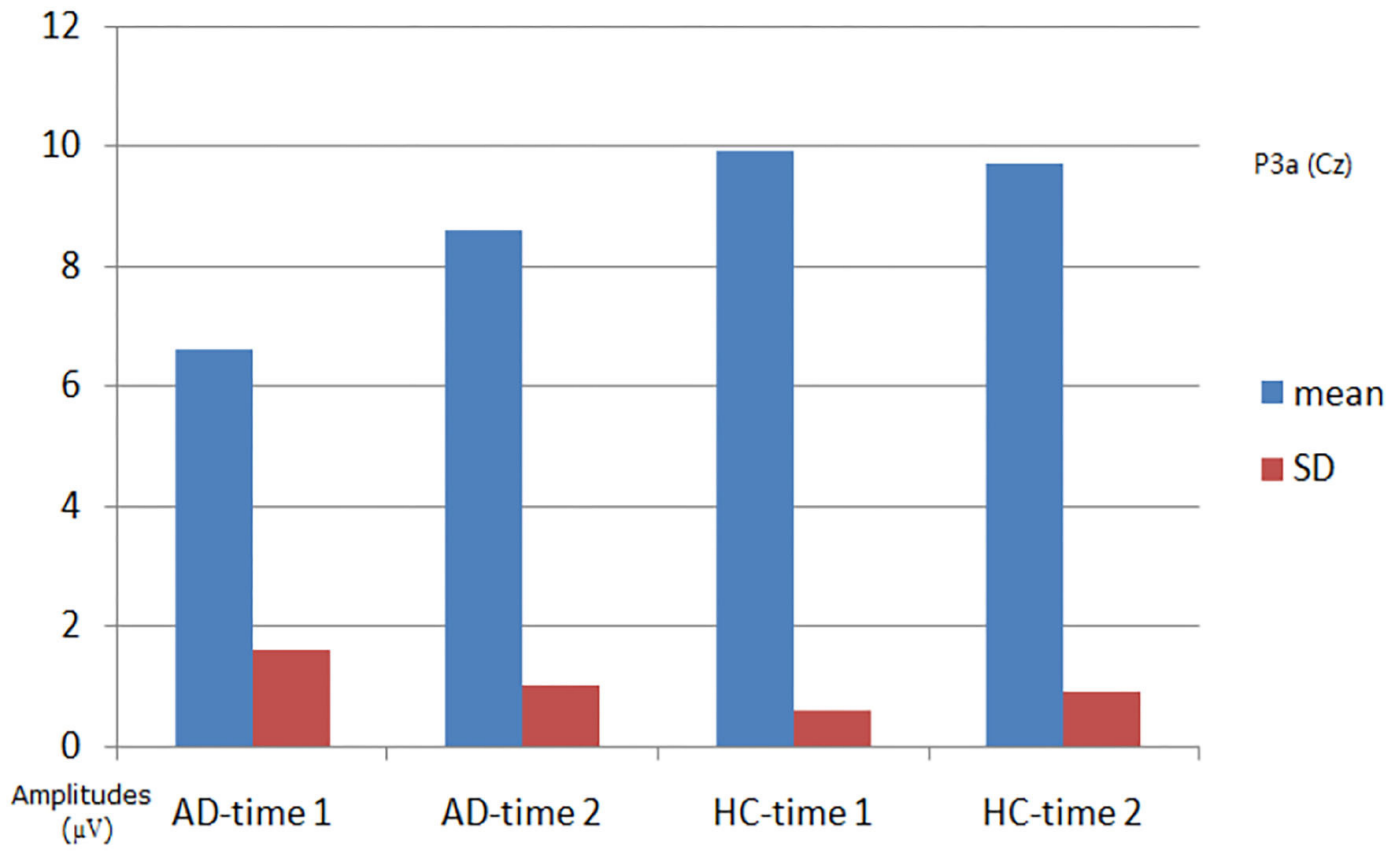
Plot of P3a amplitude analysis. The interaction effect for group × time point was significant; the simple effect for group at time 1 level and time 2 level was significant; the simple effect for time point at the alcohol dependence (AD) group level was significant; the simple effect for time point at the healthy control (HC) group level was not significant.

As shown in Figures 2, 3, for P3a amplitude, the interaction effect for group × time point was significant (*F*_1, 58_ = 38.573, *p* < 0.001). The simple effect for group at time 1 level and time 2 level was significant (*F*_1, 58_ = 107.482, 18.006, all *p* < 0.001). The simple effect for time point at AD group level was significant (*F*_1, 58_ = 40.385, *p* < 0.001). However, the simple effect for time point at HC group level was not significant (*F*_1, 58_ = 1.875, *p* = 0.181). Compared to HCs, AD patients present reductions of P3a amplitude, and after 4-week alcohol abstinence, although P3a amplitudes were improved, they were still lower than those of HCs.

For P3a latency, the interaction effect for group × time point was not significant (*F*_1, 58_ = 1.123, *p* = 0.298), and the main effect for time point was not significant (*F*_1, 58_ = 0.238, *p* = 0.630); the main effect for group was not significant (*F*_1, 58_ = 2.150, *p* = 0.153).

#### P3b Component

**Figure 4:**
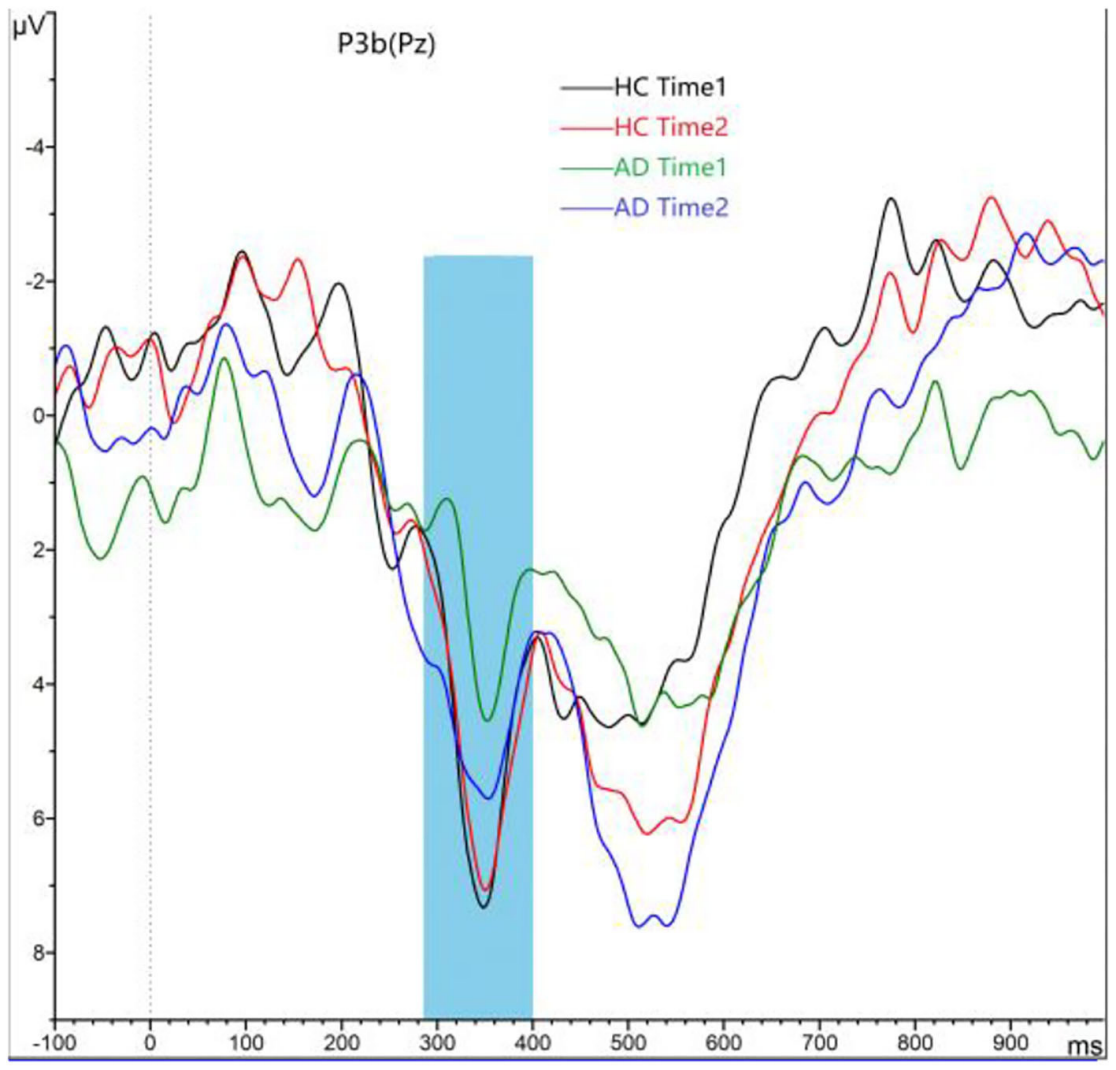
Grand averaged P3b was elicited by a three-stimulus auditory oddball paradigm for the alcohol dependence (AD) group (purple lines and green lines) and the healthy control (HC) group (black lines and red lines) at time 1 and time 2. The P3b was presented within a 280- to 650-ms latency window at the Pz electrode site. The gray area is timeframe.

**Figure 5:**
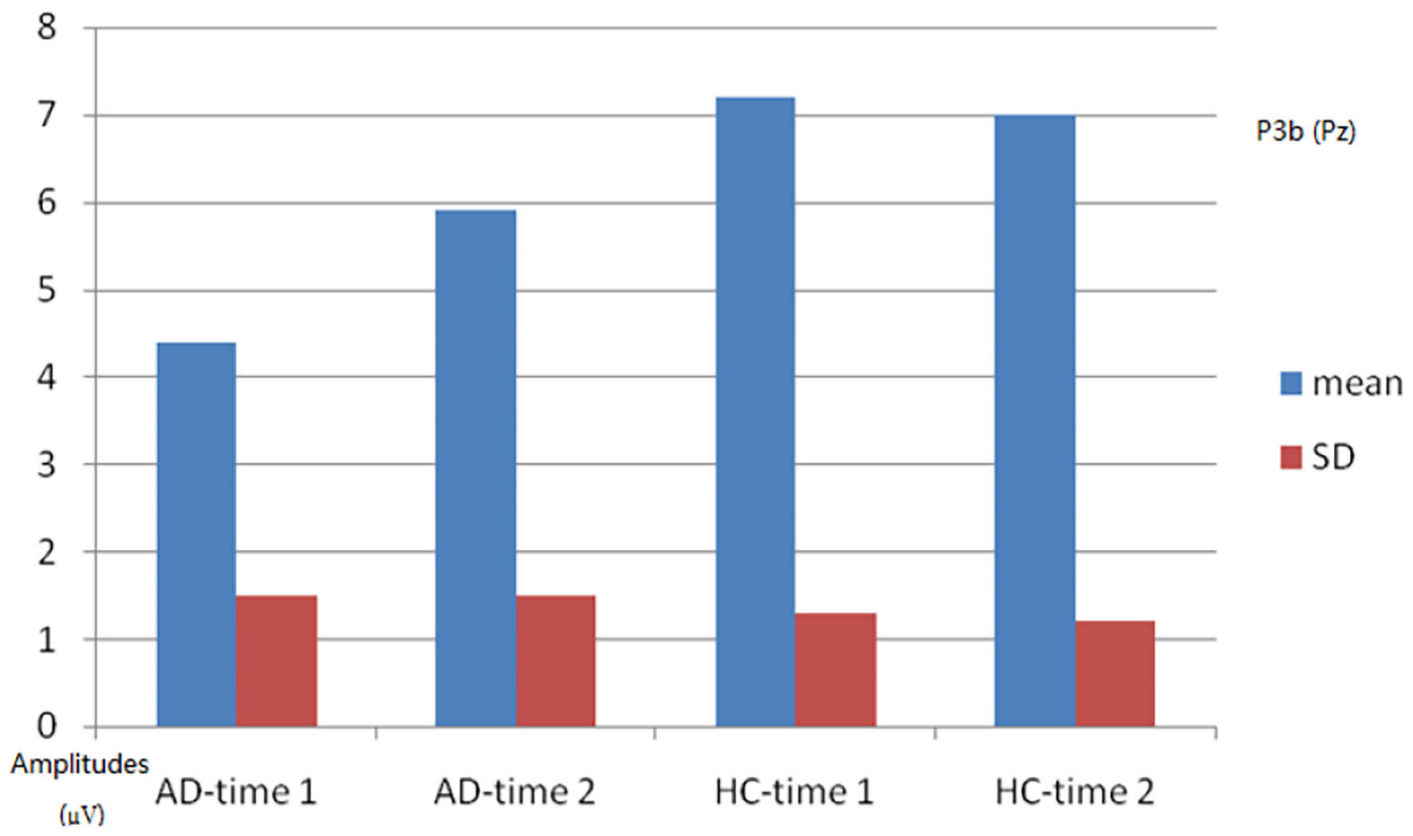
Plot of P3b amplitude analysis. The interaction effect for group × time point was significant; the simple effect for group at time 1 level and time 2 level was significant; the simple effect for time point at the alcohol dependence (AD) group level was significant; the simple effect for time point at the healthy control (HC) group level was not significant.

As shown in Figures 4, 5, for P3b amplitude, the interaction effect for group × time point was significant (*F*_1, 58_ = 10.968, *p* = 0.002). The simple effect for group at time 1 level and time 2 level was significant (*F*_1, 58_ = 56.161, 8.817, *p* < 0.001). The simple effect for time point at AD group level was significant (*F*_1, 58_ = 16.782, *p* < 0.001). However, the simple effect for time point at HC group level was not significant (*F*_1, 58_ = 0.240, *p* = 0.628). Compared to HCs, AD patients present reductions of P3b amplitude, and after 4-week alcohol abstinence, although P3b amplitudes were improved, they were still lower than those of HCs.

For P3b latency, the interaction effect for group × time point was not significant (*F*_1, 58_ = 0.046, *p* = 0.831), and the main effect for time point was not significant (*F*_1, 58_ = 0.180, *p* = 0.674); the main effect for group was not significant (*F*_1, 58_ = 0.523, *p* = 0.475).

## Discussion

This study investigated whether cognitive control deficits, which are reflected by P3a/3b deficits, in AD are present primarily just after the last alcohol intake (i.e., state-dependent) or are associated with the disorder (i.e., trait-dependent) in a longitudinal study. We compared P3a/3b amplitudes and latencies between AD patients across different stages of illness, i.e., a normal state (just after the last alcohol intake) vs. abstinence (just after 4-week abstinence). Our study results showed that compared to HCs, AD patients present reductions of P3a/3b amplitude, and after 4-week alcohol abstinence, although P3a/3b amplitudes were improved, they were still lower than those of HCs.

Many studies have manifested that the reduced P3a/3b amplitude exists in AD patients (25–29). Additionally, studies have confirmed that P300 amplitude is an endophenotype of AD risk (30, 31). Family-based association analysis shows the ACN9 gene is significantly associated with AD and P300 amplitude variation (32). Consistent with previous study findings, we discovered a relatively reduced P3a/3b amplitude in AD patients compared to HCs at a normal state (just after the last alcohol intake). In addition, this study showed that even in AD patients who appear to be rather stable in the abstinence period (just after the 4-week abstinence), P3a/3b amplitudes are still lower than those in HCs. Our results support a trait-dependent view on cognitive control deficits in AD patients, which suggests that cognitive control deficits may be a useful target for genetic studies in AD.

In support of a more state-dependent view of the illness, this study demonstrated that AD patients who stayed in the abstinence period (just after 4-week abstinence) improved in their P3a/3b amplitudes; however, those amplitudes were still lower than those of HCs. These results are in agreement with those of a previous study showing that the P3b amplitudes were significantly reduced in treatment-naive AD patients but were dramatically smaller than those observed in treated AD patients (33). Other studies have shown that the reduced P3a/3b amplitudes are no longer detectable when an internalized psychiatric comorbidity is taken into account (34). Furthermore, a previous study revealed that P3b amplitude is negatively correlated with a history of externalizing behaviors in patients with substance use disorder (35). Together with the above findings, our results indicate that cognitive control deficits are also state-dependent.

Previous studies showed that some drugs affecting substance abuse, like olanzapine or lithium, might persist in the body for more than 2 weeks and might affect the results found (36, 37). In the present study, we made a survey to all patients with AD, and they did not receive any medication for 8 weeks prior to the study; therefore, our findings were not affected by other drugs.

In conclusion, AD patients present cognitive control deficits that are reflected by ERP P3a/3b, and cognitive control deficits in AD are trait- and state-dependent. These findings suggest that improving the cognitive control function may impact the treatment effect for AD.

There are some limitations in this study. First, because of the small sample size, the study results must be considered preliminary. Future studies with larger sample sizes and the same ERP parameters are needed to verify the outcome of this study. Second, in the present study, since all participants were male, the results may be influenced by gender bias. In future research, we will consider adding female samples to verify the results of the present study. Third, owing to the deficient spatial resolution of P300, further studies with fMRI or positron emission tomography (PET) should be conducted to investigate whether cognitive control deficits in AD are trait- or state-dependent.

## Data Availability Statement

The raw data supporting the conclusions of this article will be made available by the authors, without undue reservation.

## Ethics Statement

The studies involving human participants were reviewed and approved by the Ethics Committee on Human Studies, the Affiliated Wuxi Mental Health Centre of Nanjing Medical University, Wuxi, Jiangsu Province, China. The patients/participants provided their written informed consent to participate in this study.

## Author Contributions

XL and HZ contributed to data curation, formal analysis, methodology, writing the original draft, and writing, review, and editing. XL, HZ, CJ, YX, ZZ, and JW contributed to data curation, formal analysis, and methodology. ZZ contributed to data curation, formal analysis, methodology, and writing, review, and editing. All authors contributed to the article and approved the submitted version.

## Conflict of Interest

The authors declare that the research was conducted in the absence of any commercial or financial relationships that could be construed as a potential conflict of interest.

## References

[1] Rouault M Koechlin E Prefrontal function and cognitive control: from action to language. Curr Opin Behav Sci. (2018) 21:106–11. 10.1016/j.cobeha.2018.03.008

[2] Kelley R Flouty O Emmons EB Kim Y Kingyon J Wessel JR A human prefrontal-subthalamic circuit for cognitive control. Brain. (2018) 141:205–16. 10.1093/brain/awx300 29190362 PMC5837669

[3] Schmaal L Goudriaan AE Joos L Krüse AM Dom G Van Den Brink W. Modafinil modulates resting-state functional network connectivity and cognitive control in alcohol-dependent patients. Biol Psychiatry. (2013) 73:789–95. 10.1016/j.biopsych.2012.12.025 23399373

[4] Li CS Luo X Yan P Bergquist K Sinha R. Altered impulse control in alcohol dependence: neural measures of stop signal performance. Alcohol Clin Exp Res. (2009) 33:740–50. 10.1111/j.1530-0277.2008.00891.x 19170662 PMC2697053

[5] Lim AC Cservenka A Ray LA. Effects of alcohol dependence severity on neural correlates of delay discounting. Alcohol Alcohol. (2017) 52:506–15. 10.1093/alcalc/agx015 28340213

[6] Courtney KE Ghahremani DG Ray LA. Fronto-striatal functional connectivity during response inhibition in alcohol dependence. Addict Biol. (2013) 18:593–604. 10.1111/adb.12013 23240858 PMC3683582

[7] Luck SJ Mathalon DH O'donnell BF Hämäläinen MS Spencer KM Javitt DC. A roadmap for the development and validation of event-related potential biomarkers in schizophrenia research. Biol Psychiatry. (2011) 70:28–34. 10.1016/j.biopsych.2010.09.021 21111401 PMC3116072

[8] Simonetti A Lijffijt M Kahlon RS Gandy K Arvind RP Amin P. Early and late cortical reactivity to passively viewed emotional faces in pediatric bipolar disorder. J Affect Disord. (2019) 253:240–7. 10.1016/j.jad.2019.04.076 31060010

[9] Kreusch F Quertemont E Vilenne A Hansenne M. Alcohol abuse and ERP components in Go/No-go tasks using alcohol-related stimuli: impact of alcohol avoidance. Int J Psychophysiol. (2014) 94:92–9. 10.1016/j.ijpsycho.2014.08.001 25110836

[10] Dien J Spencer KM Donchin E. Parsing the late positive complex: mental chronometry and the ERP components that inhabit the neighborhood of the P300. Psychophysiology. (2004) 41:665–78. 10.1111/j.1469-8986.2004.00193.x 15318873

[11] Polich J. Updating P300: an integrative theory of P3a and P3b. Clin Neurophysiol. (2007) 118:2128–48. 10.1016/j.clinph.2007.04.019 17573239 PMC2715154

[12] Hamidovic A Wang Y. The P300 in alcohol use disorder: a meta-analysis and meta- regression. Prog Neuropsychopharmacol Biol Psychiatry. (2019) 95:109716. 10.1016/j.pnpbp.2019.109716 31369766

[13] Cuzen NL Andrew C Thomas KG Stein DJ Fein G. Absence of P300 reduction in South African treatment-naïve adolescents with alcohol dependence. Alcohol Clin Exp Res. (2013) 37:40–48. 10.1111/j.1530-0277.2012.01837.x 22676302 PMC3491103

[14] Chen Y Norton D Mcbain R. Trait and state markers of schizophrenia in visual processing. In: Ritsner MS editor. The Handbook of Neuropsychiatric Biomarkers, Endophenotypes and Genes: Neuropsychological Endophenotypes and Biomarkers. Dordrecht: Springer Netherlands (2009). p. 211–20.

[15] Sher KJ Walitzer KS Wood PK Brent EE. Characteristics of children of alcoholics: putative risk factors, substance use and abuse, and psychopathology. J Abnorm Psychol. (1991) 100:427–48. 10.1037/0021-843X.100.4.427 1757657

[16] Glahn DC Lovallo WR Fox PT. Reduced amygdala activation in young adults at high risk of alcoholism: studies from the Oklahoma family health patterns project. Biol Psychiatry. (2007) 61:1306–9. 10.1016/j.biopsych.2006.09.041 17306772 PMC2249755

[17] Donzuso G Cerasa A Gioia MC Caracciolo M Quattrone A. The neuroanatomical correlates of anxiety in a healthy population: differences between the State-Trait Anxiety Inventory and the Hamilton Anxiety Rating Scale. Brain Behav. (2014) 4:504–14. 10.1002/brb3.232 25161817 PMC4128032

[18] Balon R. Rating scales for anxiety/anxiety disorders. Curr Psychiatry Rep. (2007) 9:271–7. 10.1007/s11920-007-0032-8 17880857

[19] Stockwell T Murphy D Hodgson R. The severity of alcohol dependence questionnaire: its use, reliability and validity. Br J Addict. (1983) 78:145–55. 10.1111/j.1360-0443.1983.tb05502.x 6135435

[20] Cheng W-J Huang M Huang P Gau Y-F Chen C-H The Chinese version of the severity of alcohol dependence questionnaire: reliability and factor structure. Taiwan J Psychiatry. (2009) 23:159–66. 10.29478/TJP.200906.0008

[21] Monaghan CK Brickman S Huynh P Öngür D Hall MH. A longitudinal study of event related potentials and correlations with psychosocial functioning and clinical features in first episode psychosis patients. Int J Psychophysiol. (2019) 145:48–56. 10.1016/j.ijpsycho.2019.05.007 31108121 PMC6988566

[22] Polich J Criado JR. Neuropsychology and neuropharmacology of P3a and P3b. Int J Psychophysiol. (2006) 60:172–85. 10.1016/j.ijpsycho.2005.12.012 16510201

[23] Comerchero MD Polich J. P3a and P3b from typical auditory and visual stimuli. Clin Neurophysiol. (1999) 110:24–30. 10.1016/S0168-5597(98)00033-1 10348317

[24] Annett M. A classification of hand preference by association analysis. Br J Psychol. (1970) 61:303–21. 10.1111/j.2044-8295.1970.tb01248.x 5457503

[25] Carlson SR Iacono WG Mcgue M. P300 amplitude in adolescent twins discordant and concordant for alcohol use disorders. Biol Psychol. (2002) 61:203–27. 10.1016/S0301-0511(02)00059-5 12385676

[26] Hill SY Jones BL Holmes B Steinhauer SR Zezza N Stiffler S. Cholinergic receptor gene (CHRM2) variation and familial loading for alcohol dependence predict childhood developmental trajectories of P300. Psychiatry Res. (2013) 209:504–11. 10.1016/j.psychres.2013.04.027 23747232 PMC3796118

[27] Hesselbrock V Bauer L O'connor S Gillen R. Reduced P300 amplitude in relation to family history of alcoholism and antisocial personality disorder among young men at risk for alcoholism. Alcohol Alcohol Suppl. (1993) 2:95–100. 7748355

[28] Hill SY Shen S. Neurodevelopmental patterns of visual P3b in association with familial risk for alcohol dependence and childhood diagnosis. Biol Psychiatry. (2002) 51:621–31. 10.1016/S0006-3223(01)01301-4 11955462 PMC3298999

[29] Chen ACH Porjesz B Rangaswamy M Kamarajan C Tang Y Jones KA. Reduced frontal lobe activity in subjects with high impulsivity and alcoholism. Alcohol Clin Exp Res. (2007) 31:156–65. 10.1111/j.1530-0277.2006.00277.x 17207114

[30] Wright MJ Luciano M Hansell NK Montgomery GW Geffen GM Martin NG. QTLs identified for P3 amplitude in a non-clinical sample: importance of neurodevelopmental and neurotransmitter genes. Biol Psychiatry. (2008) 63:864–73. 10.1016/j.biopsych.2007.09.002 17949694

[31] Polich J Pollock VE Bloom FE. Meta-analysis of P300 amplitude from males at risk for alcoholism. Psychol Bull. (1994) 115:55–73. 10.1037/0033-2909.115.1.55 8310100

[32] Hill SY Jones BL Zezza N Stiffler S. ACN9 and alcohol dependence: family-based association analysis in multiplex alcohol dependence families. Am J Med Genet B Neuropsychiatr Genet. (2015) 168b:179–87. 10.1002/ajmg.b.32295 25821040 PMC5444664

[33] Fein G Andrew C. Event-related potentials during visual target detection in treatment-naïve active alcoholics. Alcohol Clin Exp Res. (2011) 35:1171–9. 10.1111/j.1530-0277.2011.01450.x 21352244 PMC3097265

[34] Hill SY Locke J Steinhauer SR. Absence of visual and auditory P300 reduction in nondepressed male and female alcoholics. Biol Psychiatry. (1999) 46:982–9. 10.1016/S0006-3223(99)00054-2 10509181

[35] Bauer LO CNS recovery from cocaine, cocaine and alcohol, or opioid dependence: a P300 study. Clin Neurophysiol. (2001) 112:1508–15. 10.1016/S1388-2457(01)00583-1 11459691

[36] Sani G Kotzalidis GD Vöhringer P Pucci D Simonetti A Manfredi G Effectiveness of short-term olanzapine in patients with bipolar I disorder, with or without comorbidity with substance use disorder. J Clin Psychopharmacol. (2013) 33:231–5. 10.1097/JCP.0b013e318287019c 23422396

[37] Clark DC Fawcett J. Does lithium carbonate therapy for alcoholism deter relapse drinking? Recent Dev Alcohol. (1989) 7:315–28. 10.1007/978-1-4899-1678-5_16 2494686

